# Perceptions of Rehabilitation Managers on Implementation of the Home-Based Older Person Upstreaming Prevention (HOP-UP) Program: A Retrospective Qualitative Analysis

**DOI:** 10.7759/cureus.14760

**Published:** 2021-04-29

**Authors:** Alicia Naccarato, Christopher M Wilson, Sara K Arena

**Affiliations:** 1 Physical Therapy, Oakland University, Rochester, USA; 2 Rehabilitation Services, Beaumont Health, Troy, USA

**Keywords:** physical therapy, prevention, administration, falls, frailty

## Abstract

Introduction

The purpose of this study was to identify themes and concepts derived from responses of physical therapy clinical leaders related to implementing a prevention-focused, home-based older-adult program known as HOP-UP-PT (Home-Based Older Person Upstreaming Prevention Physical Therapy) into their clinical operations.

Methods

Following Institutional Review Board approval, a retrospective qualitative analysis of transcribed interviews obtained by six undergraduate students participating in the Oakland University Ideas to Business Program (I2B) was conducted. Students interviewed nine local physical therapy clinical managers throughout Michigan using 12 questions developed by content experts. Questions aimed to ascertain the perceived opportunities and barriers to implementing HOP-UP-PT into each respondent's practice setting, clinic demographics, and suggested price point of a prevention-focused continuing education. Interview data was analyzed using the constant comparative method to identify themes and concepts.

Results

Sixty-seven percent of respondents (n=6) reported practicing in an outpatient setting; 56% of respondents (n=5) indicated 50% or more of their clients were 65 years and older; and 67% of respondents (n=6) suggested a price point of $200-$500 for an eight-hour HOP-UP-PT training course. Three concepts (community involvement and partnership, administrative barriers to an innovative delivery model, and foundational physical therapy [PT] skills utilized in a novel approach) and eight themes (community altruism, referral source expansion, integrated community relationships, current payment methodology challenges, favorability of clinic setting and type, minimal additional training required, willingness to pay for certification training, and prevention-focused or upstream mindset) were identified.

Conclusion

Physical therapy clinical managers identified a willingness to expand current rehabilitation models and incorporate prevention-focused care delivery into the existing care delivery approach. However, barriers and opportunities must be addressed in advance of a program roll-out to achieve optimal outcomes and cost savings within the healthcare system.

## Introduction

The growing costs of falls and institutionalization associated with advancing age and frailty have brought about an opportunity for cost-savings within the health system for upstreaming prevention-focused programs [1]. Upstream healthcare is an approach used to identify and proactively address fundamental health issues to improve long-term health outcomes and decrease healthcare costs [2]. One program, Home-Based Older Person Upstreaming Prevention Physical Therapy (HOP-UP-PT), provides a multifactorial fall-prevention approach in the homes of older adults using a targeted population health intervention model [3]. The HOP-UP-PT program uses community-based senior centers to identify older adults with a potential risk of physical or functional decline and refers them for evaluation and prevention-focused physical therapy services in their home. These services include individualized exercise prescription, recommendations for adapting the home environment, and education aimed at optimizing health behaviors all using the skills of a licensed physical therapist (PT) [4]. In rehabilitation clinical settings (e.g., home care agencies and outpatient clinical settings) that have adopted the HOP-UP-PT program, employed physical therapists are able to engage in this preventative care model after completing an eight-hour continuing education training course surrounding fall prevention and the delivery model of HOP-UP-PT. Following certification, licensed physical therapists are able to deliver this seven-month preventative care model to community-dwelling older adults.

Positive improvements in fall risk, health, and the built environment after participating in a six-month PT-directed program have been reported by Arena et al. [3,4]. Furthermore, participants reported sustained positive benefits from the program in a post-participation survey, however, they identified an inability or unwillingness to pay out of pocket for the program [5]. While this model of delivery has potential for insurance payment, PT care in the home of older adults has generally been aimed at those identified as “homebound” [6]. Comparing the prevalence of indoor and outdoor falls, Kelsey et al. reported 77% of indoor falls occur in the older adult’s home and 23% occur in other indoor settings [7]. Integrating a home-based preventative care service in contrast to a solely clinic-based service may be essential for at-risk older adults in terms of transferring and applying knowledge, given the environmental familiarity and amount of time spent inside the home.

The program and its associated outcomes highlight distinctive opportunities for PTs to provide prevention-focused programming in an individual’s home environment as compared to a clinic-based setting, even for a patient who does not meet homebound criteria. These opportunities included the ability to recognize various social, personal, and environmental determinants of health that may be affecting an individual’s quality of life outside of the clinic setting [8]. In relation to other fall prevention programs, HOP-UP-PT offers licensed physical therapists the opportunity to provide a novel multimodal approach to preventative services not commonly utilized in the home healthcare setting and using community senior center referrals as the point of entry into the health care system.

Although there is a growing body of evidence for upstream, preventative care models, they are still not implemented widely or consistently operationalized [4]. Specifically, physical therapy-rehabilitation clinical managers’ readiness to implement a prevention-focused program into current clinical practice models alongside perceived barriers is unknown. As managers of rehabilitation organizations are key to implementing direct-care PT services, conducting a qualitative analysis of responses and inquiries posed through interview questions is warranted to identify perceived barriers and facilitators in implementing prevention-focused programs among established physical therapy rehabilitation facilities (e.g., home care agencies and outpatient clinics).

In order to ascertain the clinical facilities’ willingness to change and garner insight into existing paradigms of care, an analysis of these perceptions related to potential barriers was necessary to better understand the root of potential concerns of healthcare professionals and to best operationalize programs of this type. The purpose of this study was to identify themes and concepts derived from responses of physical therapy clinical leaders related to the implementation of a prevention-focused, home-based older-adult programming into their clinical operations.

Clinical relevance

By analyzing the perceptions of physical therapy clinical managers using data collected from primary-source interview responses during Oakland University’s (OU) Ideas to Business (I2B) program in Fall 2019, the evaluation of professional notions related to prevention-focused care models would assist in identifying opportunities and barriers. This understanding will be instrumental to navigate future integration of home-based programming with a preventative aim delivered by rehabilitation professionals.

## Materials and methods

Research design 

A retrospective qualitative analysis was completed via a review of written responses of telephone interviews from a market analysis completed as a component of the I2B program at OU (Rochester, MI). In the Fall of 2019, phone interviews were conducted by OU undergraduate health science and business students using a sample of convenience of physical therapy rehabilitation clinical managers in Michigan as a component of the I2B program design. After completion of the I2B program, these interview results were qualitatively reviewed in order to gain insight into the current environment surrounding contemporary models of rehabilitation care and the willingness and capacity of these managers to move beyond existing healthcare models to accommodate emerging initiatives. To ensure the rights and privacy of the I2B respondents were protected, Institutional Review Board (IRB) approval from OU was secured prior to beginning the retrospective analysis.

Background on Data Source

**Figure 1:**
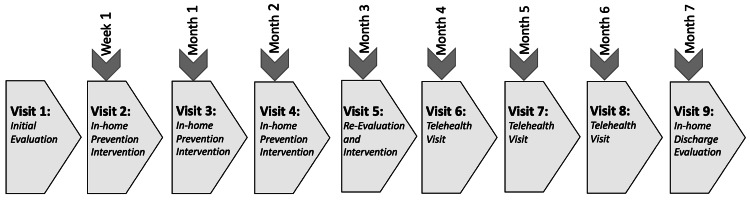
Operational Timeline of the HOP-UP-PT Program HOP-UP-PT = Home-based Older Person Upstreaming Physical Therapy. After the initial evaluation visit, the participant is seen one week later and again three weeks later in their home. The participant is seen monthly for two more in-person visits and then is transitioned to a monthly telehealth visit for three months. One month after the last telehealth visit, the participant is seen once more in the home for a discharge assessment and developing a plan to reintegrate into the community.

Oakland University's I2B program is a collaboration between undergraduate students and local business leaders to assist with market analysis and provide consultative advice aimed at strategic business implementation and commercialization. During the I2B program, six undergraduate students (three business majors and three pre-PT majors) partnered with the co-principals of the HOP-UP-PT program. After the HOP-UP-PT co-principals provided an introduction to the scientific background and clinical applicability of the program (Figure 1), the six I2B students developed a data collection approach and market analysis plan to examine the capacity to establish the program as a standard of care. The students were provided mentoring and training in the market analysis process and ultimately composed and presented their findings to an audience composed of business leaders and OU faculty.

During this market analysis, telephone interviews with physical therapy rehabilitation clinical managers were conducted to gain market insight on the perceptions of a prevention-focused physical therapy program in the homes of older adults. An informational script and interview questions were developed by the students and the principals of HOP-UP-PT describing programmatic components (interventions, training, and overall philosophy of care) and were utilized during telephone interviews administered by the six students. Participants were notified that their responses would be disseminated publicly and would be used to develop a business plan and final presentation for the I2B program; however, respondents' names and other identifying information would not be included in the dissemination. Participants were also informed that their responses would be utilized in providing a framework to complete the I2B program aims and to assist health science and business students in their future career development.

Respondent description

Nine individuals (physical therapy rehabilitation clinical managers from Michigan) were interviewed. The interviews were conducted by the aforementioned six student I2B participants. As the I2B program was not prospective research, no prior written informed consent was obtained. 

Inclusion and Exclusion Criteria

As criteria of the I2B project, individuals participating in interviews were involved in the field of physical therapy or rehabilitation, either as medical professionals or clinical leaders. Exclusion criteria of the I2B program included a respondent self-reporting they did not have an influence on business or operational decisions at their respective facility; however, no participants were excluded for this reason. No additional inclusion or exclusion criteria were applied given the retrospective nature of this study.

Data collection

Description of Phone Interview Script

After composing a list of 12 questions for the planned phone interviews (Table 1), two content experts reviewed the interview script before initiating the phone dialogues. Due to the retrospective nature of this study, no test-retest reliability or validity testing was performed on the planned questions prior to administering. Six undergraduate students (three pre-PT and three business majors) conducted the phone interviews by appointment with Michigan-based physical therapy rehabilitation clinical managers. Telephone interview appointments were scheduled on the basis of convenience for the respondents. 

**Table 1:** Interview Questions PT = physical therapist

Question Number	Question Phrasing
1	Given a brief program overview, would your organization be open to utilizing a service like this? Why or why not?
2	Do you see this as an opportunity to build new relationships and new referral sources from community partners?
3	For access to this referral system and these new older adult clients, what do you think would be an appropriate price point?
4	Do you think it should be a flat fee or scaled up or down based on the number of referrals that you accept?
5	Would you be willing to directly work with your local community centers to build a relationship with them to facilitate the older adults participating in this program? This might involve visits, talks, or health screens by you or your therapists.
6	What do you think would be an appropriate price point for therapists to become certified in this program?
7	This training program would also include education on documentation and billing to Medicare for this program. As a therapist would set up an appointment at a specific time and travel to and from the older adult’s home for approximately a 1-hour visit, do you think conventional Medicare Part B payment would sufficiently cover your costs for this service?
8	Do you currently have an option to bill Medicare part B in your organization?
9	Are there any other aspects of this evolving care model that you would like us to consider based on your personal or professional experience?
10	Which of the following best describes your PT practice setting? Home care agency Hospital Based Outpatient setting Private practice or free-standing outpatient setting Acute care hospital Other: Please describe__________________________
11	Thinking of the average age demographic of your clients, which of the following best describes the percentage of older adults (65 or older) provided care in your organization? 0-25% 26-50% 51-75% 76-100%
12	How many licensed PTs work in your setting (includes both full, part and contingent employment)?

Interview Data Collection

Interviews from nine rehabilitation clinical managers within the state of Michigan were available for analysis. Each interview required approximately 30 minutes. The I2B program participants conducted interviews in pairs with one pre-PT and one business student present during each interview.

Data analysis

Three researchers analyzed data collected for non-research purposes during OU's 2019 I2B program. The previously collected I2B written interview responses were retrospectively analyzed using qualitative research methods via the constant comparative method as described by Freeman [9]. Five phases of data analysis took place. During phase one, all interviews were de-identified by the principal investigator (PI) to protect the identity of the respondents; however, the type of facility that the respondent was managing remained identifiable (e.g., home care, hospital-based outpatient, private practice). Following de-identification, all researchers reviewed each of the nine interview responses to identify similarities and differences among participant responses to interview questions. After the initial review, the researchers met to discuss findings and to initiate a second review.

During the second phase, each researcher began coding interview results with the intent of identifying common themes and relevant supporting data corresponding to the proposed themes. Upon completion of this second review, researchers met to discuss and analyze similarities among findings and common themes found between interview responses. If a theme was not unanimously identified by all researchers, it was discarded or revised. In the third phase, the authors met again to analyze findings further and to assure all potential themes and quotes remained relevant and accurate. 

In the fourth phase, the themes and supporting data were categorized into major concepts. To increase the likelihood that appropriate themes had been captured, all three coders continued to read the interviews and meet on multiple occasions until the data was saturated and no new themes or supporting data were identified. In this phase, results were analyzed once more to control for repetition and redundancy in findings among researchers. The development of a conceptual framework model representing common themes found among rehabilitation clinical leaders’ perceptions on implementing a novel, prevention-focused clinical model was developed as an outcome of this analysis.

Finally, during phase five of data analysis, data was reviewed once more within the context of the conceptual framework, concepts, themes, and supporting data. In efforts to strengthen the internal validity of findings, researchers also searched for alternative explanations for results. In this phase, the conceptual framework underwent several revisions until investigators reached a consensus that it was an accurate representation of the data. 

## Results

Demographics

All respondents were physical therapy clinical managers from the lower peninsula of Michigan, USA. Of the respondents, 67% (n=6) described his/her practice setting as a private or freestanding outpatient setting, 11% (n=1) described his/her practice setting as a hospital-based outpatient setting, and 22% (n=2) did not provide this information. Furthermore, five respondents reported the percentage of clients over the age of 65 were as follows: less than 25% (n=1), 26-50% (n=1), 51-75% (n=4) and 76-100% (n=1). Responses were not available for the other two inquiries. 

Financial inquiries

Of the respondents, 67% felt that an appropriate price point for certification in a prevention-focused, home-based program, such as HOP-UP-PT, would range between $200 and $500 for eight hours of training. Further information regarding program certification is listed in Table 1. Additionally, 44% reported that an appropriate cost per visit for a patient’s initial evaluation for this type of program could range between $100 and $150 depending on insurance coverage. Given that HOP-UP-PT would require PTs to travel to an individuals’ dwelling to provide services, 33% of respondents expressed the need to include travel time and related expenses into the delivery model to offset these programmatic needs. 

Concepts and themes

After completing the analysis, three concepts and eight themes were identified based on the supporting data. Each is summarized in Table 2 and detailed further in the following paragraphs.

**Table 2:** Themes and Concepts PT = physical therapy

Concepts	Theme
Community involvement and partnership	Community Altruism
	Referral Source Expansion
	Integrated Community Relationships
Administrative barriers to innovative delivery model	Current Payment Methodology Challenges
	Favorability of Clinic Setting and Type
New approach using foundational PT skills	Minimal Additional Training Required
	Willingness to Pay for Certification Training
	Prevention-focused or Upstream Mindset

Community Involvement and Partnership

The first concept identified was Community Involvement and Partnerships with the supporting themes of Community Altruism, Referral Source Expansion, and Integrated Community Relationships. Respondents voiced that Community Altruism was important to their public service intentions. Specifically, the altruistic intention was summarized in the comment “We always try to get involved in the community. Sometimes they just need a check-in, who knows how much turn out you’ll actually get though” - Participant 9. Additionally, one respondent stated that “There is benefit in prevention care prior to injury and helping healthcare costs and chronic disease prevention” - Participant 3.

Referral Source Expansion was a second theme identified within the concept of Community Involvement and Partnerships. Responses included “[Finding] someone at risk and sending [PTs] to them is a very realistic entry point to the healthcare system” - Participant 5 and “We’ve done senior expos before with community centers - it’s a definite resource to get your name out there, and I like the 10-mile radius for the community aspect of it as well” - Participant 2. This demonstrated that some respondents and their organizations have previously identified this as an opportunity for new referrals, and that concrete steps had been taken, and yet further opportunities were warranted.

Finally, Integrated Community Relationships was the third theme identified within the concept of Community Involvement and Partnerships. Supporting data included responses such as, “People want to meet PTs and form that relationship, even if they don’t need care” - Participant 2 and “Sometimes I have people who don’t have anything wrong and just want to be ‘checked out’ by me for the reassurance after they’ve met me. Just the presence of someone else seeing you and word of mouth is great. The PT going to the actual facility is a benefit” - Participant 2. This supporting data provides evidence that PTs already have a role as a valuable healthcare provider in the community and are sought out for professional advice; however, a limited overall consistency or organization within these circumstantial relationships may also be present.

Administrative Barriers to an Innovative Delivery Model

A second concept identified was the presence of Administrative Barriers to an Innovative Delivery Model with the theme of Current Payment Methodology Challenges. Furthermore, it was found that this approach would have Favorability of Clinic Setting and Type when providing a prevention-focused innovative delivery model. The theme of Current Payment Methodology Challenges was supported by respondents’ quotes including “This would be tough to have the reimbursement of Medicare part B cover your visit due to additional travel time” - Participant 2 and “We would not be able to bill for time traveled” - Participant 3. One respondent additionally stated “Drive time and everything? There would typically be a drive time allowance on top of the reimbursement” - Participant 8, also highlighting a potential cost burden unaddressed in current rehabilitation outpatient payment models.

Furthermore, as respondents indicated that this type of program may have Favorability of Clinic Setting and Type, comments included, “Clinics that are looking to increase patient population would definitely want to utilize this program” - Participant 2, “Smaller clinics have a lot more availability to go ahead with programs as such, whereas chain business may have a harder time getting involved in programs” - Participant 9 and “....in outpatient and homecare, [the program] would be very important”- Participant 6. Supporting data also provided insight into the types of practice settings that would be of most benefit. For example, respondents noted that a program like HOP-UP-PT would be of most use in clinics “...specifically with geriatric PTs” - Participant 5 and that among the population of older adults remaining active in their communities but also at risk for decline due to home-based factors,“...it would be good to get [involved]” - Participant 1.

New Approach Using Foundational PT Skills

Investigators identified a third concept, presented within similar programs to HOP-UP-PT, a New Approach using Foundational PT Skills, which represented themes of Minimal Additional Training Required, a Willingness to Pay for Certification Training, and the need for providers to have a Prevention-focused or Upstream Mindset.

Regarding the theme of Minimal Additional Training Required, one respondent stated, “For a PT to do this prevention, it doesn’t require a license to do prevention. All PTs should be certified to do prevention” - Participant 4. For the theme of Willingness to Pay for Certification Training, respondents stated, “Maybe $250 to get the certification to be distinguished. You’d need to do a lot of testing to see what kind of certification you would be providing to PTs, because all PT’s should be certified and should know how to do fall-prevention” - Participant 4 and “There’s so much con-ed out there. Anywhere between $75-80 an hour. $100-150 an hour for online” - Participant 1. Another respondent discussed options for online training and suggested, “PT is a hands-on profession; therefore, it is essential for live training... Lots of online seminars are popular nowadays” - Participant 6. Finally, the theme of a Prevention-focused or Upstream Mindset of these clinical managers was supported by the statement, “There is benefit in preventing care prior to injury and help healthcare cost and chronic disease prevention” - Participant 3.

## Discussion

**Figure 2:**
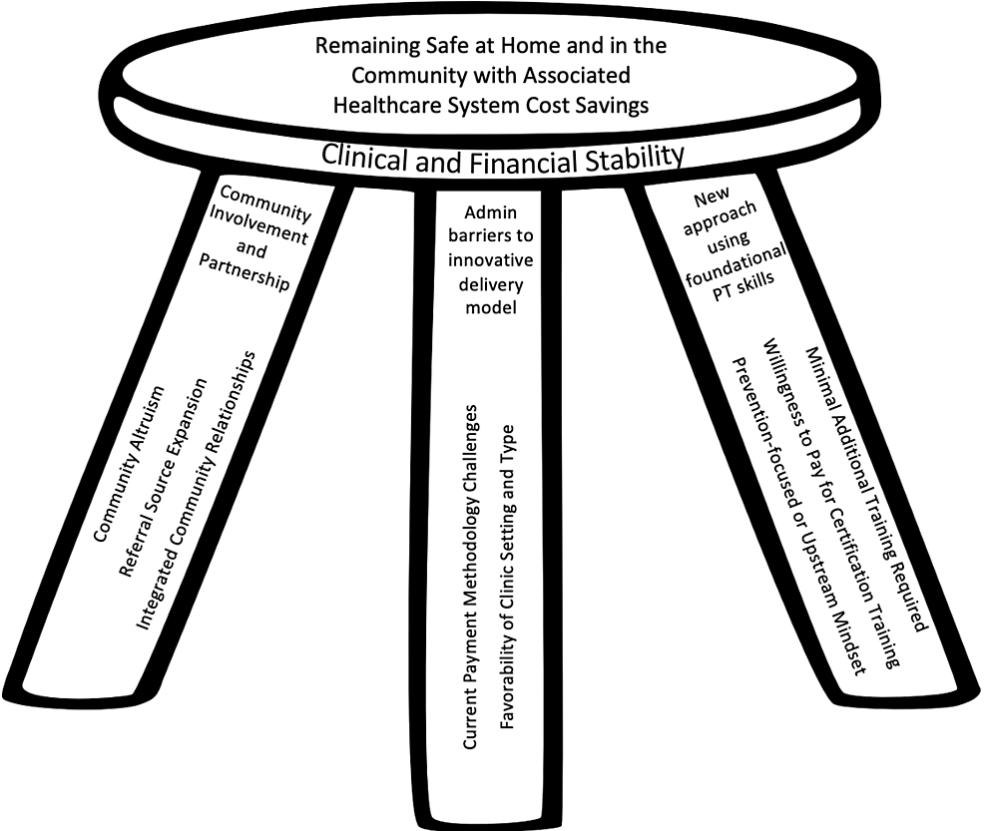
Conceptual Framework of Clinical Managers’ Perceptions of Integration of Prevention-Focused Home-Based Services for Older Adults PT = physical therapy

The purpose of this study was to identify themes and concepts derived from responses of physical therapy rehabilitation clinical leaders related to the implementation of a prevention-focused, home-based older-adult program into their clinical operations. These responses were centered around the need for primary prevention in relation to the traditional rehabilitative practice models of PT care. Figure 2 depicts a conceptual framework using the themes and concepts identified in this analysis. As investigators determined that all of the relevant concepts and related themes were critical in implementing this treatment paradigm into rehabilitation operations, a three-legged stool was chosen to depict these aspects. The three-legged stool emphasizes the need for a solid foundation when looking to provide effective care (for when one leg is removed, the stool becomes unstable) and therefore serves as a relevant symbol for the multifaceted approach required during the implementation of preventative services. Specifically, perceived barriers and facilitators identified by rehabilitation clinic managers, a readiness to implement a novel prevention program into current rehabilitative models, and expanded opportunities for wellness in at-risk older adults and healthcare professionals through prevention-focused programming were identified and are depicted in Figure 2. 

A broad-reaching need to have older adults remain safe in their homes and communities with the added benefit of healthcare cost savings stands at the core of a targeted population health program, including the HOP-UP-PT program; however, the clinical and financial sustainability of these programs must be considered [1]. This study identified perceptions of respondents in implementing a program of this type, including the barriers and opportunities. Establishing the respondents’ willingness to expand opportunities for community engagement was significant in terms of distinguishing the long-term motivation and ambitions of clinical leaders. Furthermore, administrative barriers to implementation of a prevention-focused program included protocol training, payment methodology, accounting for travel time, and integration within current clinical operations. These must be mitigated in order to establish sustainable prevention-focused approaches that have clinical relevance and long-term opportunity for health professionals and at-risk older adults [10].

The findings of this study are in congruence with prior related investigations. Kiami et al. reported that the primary barrier to participating in fall prevention programs was participants’ perceptions that their fall risk was not high enough to warrant action [11]. Additional barriers commonly described by the study population included accessibility, denial, fear of pain with exercise, and inconvenience [11]. The HOP-UP-PT program spans a timeframe of seven months; Schoberer and Breimaier found that fall intervention programs conducted for longer than a six-month period showed a significant reduction in falls in long-term care facilities despite the perceived barriers [10]. Walker et. al. also noted that older adults who had not yet fallen experienced benefits from early preventative fall reduction strategies as the number of falls per year within their intervention dropped from 40 to 35 in their pretest/posttest setting, which is consistent with the perceptions of our respondents [12]. 

Identifying and describing various pre-existing perceptions of implementing a preventative care program into the current care system offered insight into the integration of future innovations in healthcare, leading to a reduction in falls and empowerment of older adults to remain safe and active in their own homes with associated cost-savings likely to follow. Each interview respondent demonstrated a willingness to provide quality health services, diversify referral sources, and to increase community outreach for their respective facilities. In addition, as interview respondents reported serving patients in home care, outpatient, and private and hospital-based practice settings, the findings may be applicable to different clinical settings and operations. Addressing barriers regarding travel time reimbursements, ideal practice settings for implementation, and an upstream mindset is important for the implementation of novel initiatives like HOP-UP-PT in order to positively impact older adults, their local communities, and healthcare facilities.

Initiating novel models with an upstream, prevention focus aligns with the American Physical Therapy Association’s (APTA) Council on Prevention, Health Promotion, and Wellness in Physical Therapy mission to “develop and disseminate best practices in prevention, health promotion, and wellness for all individuals and populations" [13]. The APTA also identifies the knowledge base, skills, and abilities present within PTs to guide people toward optimal health and well-being, including a focus on facilitating behavior change to improve health outcomes [13]. Implementing models that aim to prevent falls before they occur will also aid in the establishment of long-term relationships and connections between older adults and PTs, where the PTs become a reliable source of motivation, encouragement and model of health for others [13,14]. 

Study limitations 

Limitations to this study included using a retrospective sample of convenience, which may have introduced bias into the results. In addition, there may have been a limited accuracy of quotes as the interviews were not audio recorded. As all respondents were from the lower peninsula of Michigan, USA, generalizability to other regions may be limited. Additionally, the small sample size is a limitation. It is noted these interviews were conducted prior to the massive societal shift to a virtual educational content delivery and healthcare service disruption from the COVID-19 pandemic; therefore, it is unclear if respondents’ views would be affected by these new circumstances. Due to the retrospective nature of this study, a lack of test-retest reliability could have affected study outcomes. Specifying sex, gender, age, or other demographic information was not the primary purpose of I2B research and thus was not collected. 

Future research

Future research aiming to increase diversity among clinic manager demographics in terms of geographical location and practice setting may strengthen generalizability among rehabilitation clinic leaders. Additionally, a study analyzing a larger sample size for interview responses would be beneficial in identifying potential perceptions of rehabilitation clinic leaders not yet addressed. 

## Conclusions

Physical therapy clinic managers identified a willingness to expand current rehabilitation models and incorporate prevention-focused care delivery into the existing care delivery approach. They identified that this approach would improve the functioning and health of the older adult population and that PTs were well suited to provide these services clinically. Respondents identified 1) a desire to have community involvement and partnership, 2) administrative barriers to this innovative delivery model, and 3) that this new approach to care would utilize foundational PT skills. In order to achieve the goal of prevention-focused, holistic services administered by PTs for at-risk older adults, these barriers and opportunities must be addressed in advance of a program roll-out to achieve optimal outcomes and cost savings within the healthcare system.
